# Twin introductions by independent invader mussel lineages are both associated with recent admixture with a native congener in Australia

**DOI:** 10.1111/eva.12857

**Published:** 2019-09-18

**Authors:** Iva Popovic, Ambrocio Melvin A. Matias, Nicolas Bierne, Cynthia Riginos

**Affiliations:** ^1^ School of Biological Sciences University of Queensland St Lucia Qld Australia; ^2^ Institute of Biology University of the Philippines Diliman Quezon City Philippines; ^3^ Institut des Sciences de l’Evolution UMR 5554 CNRS‐IRD‐EPHE‐UM Université de Montpellier Montpellier France

**Keywords:** demographic history, hybrid, introgression, marine invasions, mussels, *Mytilus*, non‐native species, transcriptome

## Abstract

Introduced species can impose profound impacts on the evolution of receiving communities with which they interact. If native and introduced taxa remain reproductively semi‐isolated, human‐mediated secondary contact may promote genetic exchange across newly created hybrid zones, potentially impacting native genetic diversity and invasive species spread. Here, we investigate the contributions of recent divergence histories and ongoing (post‐introduction) gene flow between the invasive marine mussel, *Mytilus galloprovincialis,* and a morphologically indistinguishable and taxonomically contentious native Australian taxon, *Mytilus planulatus*. Using transcriptome‐wide markers, we demonstrate that two contemporary *M. galloprovincialis* introductions into south‐eastern Australia originate from genetically divergent lineages from its native range in the Mediterranean Sea and Atlantic Europe, where both introductions have led to repeated instances of admixture between introduced and endemic populations. Through increased genome‐wide resolution of species relationships, combined with demographic modelling, we validate that mussels sampled in Tasmania are representative of the endemic Australian taxon (*M.* *planulatus*), but share strong genetic affinities to *M. galloprovincialis*. Demographic inferences indicate late‐Pleistocene divergence times and historical gene flow between the Tasmanian endemic lineage and northern *M. galloprovincialis*, suggesting that native and introduced taxa have experienced a period of historical isolation of at least 100,000 years. Our results demonstrate that many genomic loci and sufficient sampling of closely related lineages in both sympatric (e.g. Australian populations) and allopatric (e.g. northern hemisphere *Mytilus* taxa) ranges are necessary to accurately (a) interpret patterns of intraspecific differentiation and to (b) distinguish contemporary invasive introgression from signatures left by recent divergence histories in high dispersal marine species. More broadly, our study fills a significant gap in systematic knowledge of native Australian biodiversity and sheds light on the intrinsic challenges for invasive species research when native and introduced species boundaries are not well defined.

## 1 INTRODUCTION

The ability of introduced species to alter the ecology and evolution of native communities is a fundamental issue for understanding the long‐term impacts of biological invasions (Colautti & Lau, 2015; Rius, Turon, Bernardi, Volckaert, & Viard, 2015). When introduced species are distinct in morphology, life history or ecology from native residents, studies have documented profound effects on receiving communities at multiple levels of biological organization. Successful invaders may directly or indirectly displace native species though predation or competition (e.g. Arcella, Perry, Lodge, & Feder, 2014; Branch & Steffani, 2004), inflict damage to local habitats (e.g. Robinson, Griffiths, Branch, & Govender, 2007) and prompt cascading community‐level impacts that can transform entire ecosystems (e.g. Griffiths, Hockey, Erkom Schurink, & Roux, 1992; Shine, 2010). From a molecular perspective, native and introduced species that have been isolated for short periods of time or have experienced historical contact throughout their evolutionary histories may retain genomes semi‐permeable to gene flow (Roux et al., 2016). If native and introduced taxa remain reproductively semi‐isolated, secondary contact can promote ongoing genetic exchange across hybrid zones, imposing less discernible, but potentially severe genetic impacts that can result in complex evolutionary outcomes for endemic populations (Ellstrand & Schierenbeck, 2000).

In the absence of complete reproductive barriers, introgression may promote successful introductions through the spread of locally favoured variants into introduced genomic backgrounds (Hovick & Whitney, 2014; Schierenbeck & Ellstrand, 2009). Hybridization may also impede invasions by trapping species barriers at environmental boundaries (Bierne, Welch, Loire, Bonhomme, & David, 2011; El Ayari, Menif, Hamer, Cahill, & Bierne, 2019; Kovach et al., 2016). Conversely, introgression into native genomic backgrounds may alter native genetic diversity (Blum, Walters, Burkhead, Freeman, & Porter, 2010; Fitzpatrick et al., 2010; Todesco et al., 2016) or eliminate parental genotypes entirely through introgression swamping (Arcella et al., 2014; Glotzbecker, Walters, & Blum, 2016; Riley, Bradley Shaffer, Randal Voss, & Fitzpatrick, 2003). Despite potentially significant consequences for endemic diversity and invasive species spread, hybrid invasions are likely to go undetected if native and introduced species boundaries are not well defined (Geller, Darling, & Carlton, 2010). Indeed, comparative genomic studies have revealed a high occurrence of weakly differentiated and semi‐reproductively isolated species within the “grey zone” of the speciation continuum naturally occurring in both terrestrial and marine systems (De Queiroz, 2007; i.e. 0.075–2% average transcriptome‐wide molecular divergence; Roux et al., 2016), highlighting taxonomic issues pertinent for delineating closely related lineages (Galtier, 2019). For invasive species research, however, the “grey zone” raises additional challenges for detecting species introductions and understanding the outcomes of secondary contact. Furthermore, when hybridization is possible between native and introduced taxa, genetic tools and multilocus genotyping become essential for resolving the consequences of hybridization for endemic populations (Viard, David, & Darling, 2016).

In the marine environment, semi‐reproductively isolated species complexes are a common and persistent issue for detecting marine invasions (Bouchemousse, Liautard Haag, Bierne, & Viard, 2016; Viard et al., 2016). Many marine species exhibit high fecundity and dispersal potential (through planktonic larvae) that support elevated rates of gene flow and low genetic differentiation between populations (Gagnaire et al., 2015). Weak differentiation is also sustained by large effective population sizes that slow down genetic drift, such that high levels of ancestral polymorphisms are common features of many diverging marine taxa (e.g. Fraïsse, Belkhir, Welch, & Bierne, 2016; Gagnaire, Normandeau, & Bernatchez, 2012). Genomic methods based on differentiation (i.e. *F*‐statistics) alone may fail to distinguish between recently diverged native and introduced species or identify sources of introduced populations (Tepolt, 2015; Viard et al., 2016). Furthermore, because lineages with large effective populations sizes are not expected to reach genome‐wide reciprocal monophyly for many generations (up to 10N_e_ generations; Keightley & Eyre‐Walker, 2012), both incomplete lineage sorting and recent (post‐introduction) gene flow may lead to shared polymorphisms between semi‐isolated species (Marko & Hart, 2011). Ongoing introgression is therefore difficult to recognize and quantify when native and introduced taxa show either weak divergence or genomes shaped by complex speciation histories of intermittent historical contact (Fraïsse et al., 2016). Additionally, because both *F*_ST_ and linkage disequilibrium‐based population clustering approaches assume a mutation‐drift equilibrium and a single demographic model (i.e. Wright's island model; Wright, 1951), such methods cannot provide explicit tests of migration or demographic history underlining patterns of genetic ancestry (Patterson et al., 2012; Pickrell & Pritchard, 2012). In turn, neglecting complex demographic scenarios that have shaped the genetic backgrounds of closely related taxa may mislead interpretations of population relationships and introgression between endemic and introduced marine populations (Rougemont & Bernatchez, 2018).

Methods that model demographic histories across many loci offer powerful approaches for resolving the contributions of ancestral polymorphism and recent introgression to shared variation between species (Fagundes et al., 2007; Fu & Li, 1997; Pritchard, Seielstad, Perez‐Lezaun, & Feldman, 1999). Coalescent genealogy samplers, for example, allow explicit inferences of divergence and migration rate parameters (e.g. isolation‐with‐migration models; Hey & Nielsen, 2004, 2007; Kuhner, 2009; Marko & Hart, 2012; Sousa, Carneiro, Ferrand, & Hey, 2013), but rely on full‐likelihood calculations that are computationally intractable for large genomic data sets or complex demographic histories experienced by marine taxa (Roux et al., 2016). Approximate Bayesian computations (ABC) allow tests of alternative divergence models and rely on few samples per taxon for robust inferences of divergence histories while avoiding full‐likelihood computations (Beaumont, 2010; Beaumont, Zhang, & Balding, 2002; Bertorelle, Benazzo, & Mona, 2010; Pritchard et al., 1999; Roux et al., 2016). ABC approaches have been highly informative for reconstructing invasion routes (Barker, Andonian, Swope, Luster, & Dlugosch, 2017; Estoup & Guillemaud, 2010; Lombaert et al., 2011) and identifying introgression in contact zones (Estoup, Beaumont, Sennedot, Moritz, & Cornuet, 2004; Estoup, Wilson, Sullivan, Cornuet, & Moritz, 2001; Guillemaud, Beaumont, Ciosi, Cornuet, & Estoup, 2010; Pascual et al., 2007; Roux, Tsagkogeorga, Bierne, & Galtier, 2013). Coalescent approximations can also strengthen comparative inferences of historical relationships between weakly differentiated introduced and native taxa when taxonomic boundaries are also challenged by invasive introgression.

Marine mussels in the genus *Mytilus* are a compelling example of a morphologically cryptic and reproductively semi‐isolated group of species that have also experienced complex evolutionary histories of past hybridization and contemporary human‐mediated secondary contact. The Mediterranean native, *M. galloprovincialis,* is recognized as one of the world's most widespread invasive species and is surprisingly the only *Mytilus* congener known to pose invasion threats globally (Lowe, Browne, Boudjelas, De, & Poorter, 2000; McDonald, Seed, & Koehn, 1991). Despite several pre‐ and postzygotic reproductive isolating mechanisms between *Mytilus* species (e.g. Bierne, Bonhomme, Boudry, Szulkin, & David, 2006; Bierne, Bonhomme, & David, 2003; Bierne, Borsa, et al., 2003; Skibinski, Beardmore, & Cross, 1983), *M. galloprovincialis* has a well‐documented history of hybridizing with native congeners where their ranges overlap throughout its introduced distribution in the northern hemisphere (e.g. Japan, Brannock, Wethey, & Hilbish, 2009; California, Rawson, Agrawal, & Hilbish, 1999; Saarman & Pogson, 2015). There is also strong evidence for differential introgression with sister species, *Mytilus edulis,* in some parts of its present day native range across mosaic hybrid zones in Europe (Bierne, Borsa, et al., 2003; Fraïsse et al., 2016; Fraïsse, Roux, et al., 2018; Gosset & Bierne, 2013; Rawson & Hilbish, 1998; Roux et al., 2014). Interspecific admixture with *M. edulis* has subsequently led to pronounced genetic differentiation between *M. galloprovincialis* lineages from the Mediterranean Sea and Atlantic Europe (Fraïsse et al., 2016; Quesada, Wenne, & Skibinski, 1995). These divergent *M. galloprovincialis* lineages display partial reproductive isolation (El Ayari et al., 2019) and have both been implicated in independent invasions into California (Daguin & Borsa, 2000; McDonald & Koehn, 1988) and South Africa (Branch & Steffani, 2004), respectively.

Despite a number of genetic investigations, less is known about the invasive distribution of *M. galloprovincialis* in other parts of the southern hemisphere (Daguin & Borsa, 2000; Gardner, Zbawicka, Westfall, & Wenne, 2016; Gérard, Bierne, Borsa, Chenuil, & Féral, 2008; Hilbish et al., 2000; Larraín, Zbawicka, Araneda, Gardner, & Wenne, 2018; McDonald et al., 1991; Oyarzún, Toro, Cañete, & Gardner, 2016). The widespread occurrence of northern *M. galloprovincialis* haplotypes along temperate coastlines in Chile, New Zealand and Australia suggests that introduced populations are established in coastal regions (e.g. Gardner et al., 2016; Larraín et al., 2018; Westfall & Gardner, 2010). However, the existence of morphologically cryptic *Mytilus* lineages endemic to the southern hemisphere has sustained ongoing confusion regarding *M. galloprovincialis* introductions in these regions (Ab Rahim et al., 2016; Colgan & Middlefart, 2011; Dias, Fotedar, & Snow, 2014; Larraín et al., 2018; Westfall & Gardner, 2010). In Australia, fossil *Mytilus* shells predating European contact (New South Wales, Donner & Jungner, 1981; South Australia, Hope, Lampert, Edmondson, Smith, & Tets, 1977; Tasmania, Colhoun, Turner, & Van de Geer, 1982; reviewed in McDonald et al., 1991; Hilbish et al., 2000) support an endemic taxon. Yet, resolving the taxonomic affinity of the native Australian species, originally named *Mytilus planulatus* Lamarck 1819, has been complex: initial genetic studies using size polymorphic nuclear markers suggested high genetic similarity to northern *M. galloprovincialis* and described the native taxon as an endemic southern hemisphere lineage of *M. galloprovincialis* (Borsa, Daguin, & Bierne, 2007; Daguin & Borsa, 2000; McDonald et al., 1991)*.* Later phylogenetic comparisons of the mitochondrial marker COI, however, dated the origins of southern *Mytilus* to the late Pleistocene approximately 0.84 (0.5–1.3 mya, Gérard et al., 2008) and 1.2 million years ago, implicating deeper historical isolation between northern and southern taxa (Hilbish et al., 2000).

To date, taxonomic delineation of the Australian endemic taxon has been hampered by a limited number of reliable loci that has also precluded detailed investigations of its recent evolutionary history. In particular, high levels of ancient incomplete lineage sorting among *Mytilus* species (resulting in various gene topologies) are likely to obscure signals of present day introgression and further amplify discordant species relationships when few loci are examined (Fraïsse, Haguenauer, et al., 2018). Thus, it remains unresolved (a) whether Australian native *Mytilus* comprise a lineage sufficiently divergent from northern *M. galloprovincialis* to warrant species status as *M. planulatus*, and (b) whether the history of Australian mussels reflects recent divergence only, or includes ongoing hybridization with introduced congeners. Here, we use transcriptome‐wide population genomic analyses with ABC inferences to test alternative hypotheses regarding the origins of Australian *Mytilus* mussels (hereafter referred to as its current nomenclature, *M. planulatus*) and resolve the contributions of past and ongoing (post‐introduction) gene flow with introduced *M. galloprovincialis* in south‐eastern Australia. This study represents the first transcriptome‐wide investigation of demographic history and introgression between introduced and Australian endemic *Mytilus* species.

## 2 METHODS

### 2.1 Sample collection, RNA extraction and sequencing

Mussels were collected from wild populations from rocky intertidal or subtidal environments (Table 1). Outgroup specimens (*Mytilus californianus*, *n* = 3; *Mytilus trossulus*, *n* = 3; *M. edulis n* = 3) were collected from known contemporary allopatric ranges to minimize the possibility of sampling hybrid individuals. We collected Atlantic (*n* = 5) and Mediterranean (*n* = 10) *M. galloprovincialis* belonging to two genetically divergent lineages separated by the Almeria‐Oran front, including a population east of the Siculo–Tunisian Strait dividing the eastern and western Mediterranean (Fraïsse et al., 2016; Table 1). In Australia, we targeted previously unsampled locations to include populations where introductions would be likely, such as large shipping harbours (i.e. Sydney Harbour, *n* = 9) and a second location (i.e. Batemans Bay, *n* = 9), to extend previous sampling efforts across the eastern coast of Australia. We additionally included samples from Tasmania (*n* = 5), where high frequencies of the divergent southern mitochondrial haplotypes have been reported (Colgan & Middlefart, 2011). Individuals were genotyped for the species diagnostic marker *Glu‐5’* (Rawson, Joyner, Meetze, & Hilbish, 1996) to obtain a first clue about species identity. Preliminary assignment of Australian samples (total samples = 23; Table 1) as *M. planulatus* was based on the F‐type (female) mitochondrial marker COIII using primers from Riginos, Hickerson, Henzler, and Cunningham (2004) and phylogenetic analyses using neighbour‐joining statistics implemented in Geneious 8.1. Total RNA was extracted from 10 to 20 mg of mantle tissue (preserved in RNAlater) using the RNeasy Plant Mini Kit and following the animal tissue protocol with an additional DNAse treatment to remove genomic DNA. Individual cDNA libraries were constructed and barcoded using the TruSeq stranded mRNA kit (Illumina), with average insert sizes of 250–300 bp. Paired‐end (125 bp fragments) libraries were sequenced across three lanes of an Illumina Hiseq2000 or across a single lane of an Illumina Hiseq4000.

**Table 1:** Details of samples and collection locations

Taxon	Sampling location	Range	Individuals sequenced
*Mytilus californianus*	Scripps Institute of Oceanography, California, USA	Native	3
*Mytilus trossulus*	Lighthouse Park, British Columbia, Canada	Native	3
*Mytilus edulis*	Darling Marine Station, Maine, USA	Native	3
*Mytilus galloprovincialis*	Primel, France (Atlantic)*	Native	5
	Crique, Les Issambres, France (Mediterranean‐West)*	Native	5
	Herceg Novi, Montenegro (Mediterranean‐East)*	Native	5
***Mytilus galloprovincialis***	**Sydney Harbour, New South Wales, Australia** a	**Introduced** a	**9**
	**Batemans Bay, New South Wales, Australia** a	**Introduced** a	**9**
***Mytilus planulatus***	**Spring Bay, Tasmania, Australia**	**Native**	**5**

Note

Samples in **bold** indicate species identity (based on genome‐wide analyses) and range. Populations marked with an asterisk were used to construct a de novo reference transcriptome assembly for *M. galloprovincialis*.

a *M. galloprovincialis* samples are introgressed with *M. planulatus.*

RNA‐seq data sets were trimmed and filtered to select for the highest quality reads. Three native range *M. galloprovincialis* populations (Table 1) were used to construct a de novo reference transcriptome assembly free of contaminant sequences. Details regarding RNA‐seq read filtering, processing and de novo transcriptome assembly are outlined in the Appendix S1. The resulting 159,985 nuclear sequences were used as a reference assembly for variant discovery and as input for all downstream analyses.

### 2.2 Genomic data filtering

RNA‐seq reads from Australian samples and four northern *Mytilus* taxa were mapped to the *M. galloprovincialis *de novo reference transcriptome (Appendix S1) using Bowtie2 (Langmead & Salzberg, 2012), and PCR duplicates were removed using Picard MarkDuplicates (http://picard.sourceforage.net). Different subsequent analyses required different filtering schemes (Figure S1): for genomic analyses (with the exception of ABC inference), single nucleotide polymorphisms (SNPs) were called using Freebayes (https://github.com/ekg/freebayes) and filtered in VCFtools (Danecek et al., 2011). Variant sites below a minimum genotype quality of 30 and a minimum mean depth coverage of 10 reads were excluded. For principal component analyses and genomic analyses of species relationships (network analysis and topology weighting), we removed singletons, indel variants and positions with missing data. Genotypes were statistically phased using beagle v4.1 (Browning & Browning, 2007). We generated consensus sequences for individual haplotypes using the corresponding VCF file and reference assembly in BCFtools v1.3.1. For analyses investigating population structure and admixture (ADMIXTURE and TreeMix), we additionally removed SNPs with a minor allele frequency of less than 5%, but retained positions with up to 20% missing data. For variant calling and filtering pertaining to ABC inference, refer to *Approximate Bayesian Computations (ABC) of demographic history.*

### 2.3 Analyses of population structure and admixture

We first established whether individuals sampled in Australia belonged to introduced or endemic genomic backgrounds by comparing Australia mussel genotypes against northern native range *M. galloprovincialis.* Principal component analysis and genomic clustering analyses using the program ADMIXTURE (Alexander, Novembre, & Lange, 2009) were undertaken to ascertain the presence of the putative endemic *M. planulatus* and the possibility of admixture between native and introduced populations. In ADMIXTURE, we estimated individual ancestry proportions with *M. edulis* as an outgroup taxon. VCFtools and PLINK v1.90 (Purcell et al., 2007) were used to convert the filtered VCF output to BED format files as input, which reduced the original data set to 34,097 biallelic SNPs across 3,945 contigs. We ran ADMIXTURE with 100 iterations and used the cross‐validation procedure with 50 replicates for *K* = 1 to *K* = 10 genetic clusters.

Second, to test for introgression and to validate potential sources of gene flow, we performed joint analyses of migration and historical population relationships in TreeMix v1.12 (Pickrell & Pritchard, 2012). TreeMix uses allele frequency correlations between populations to infer a maximum‐likelihood population tree representative of the phylogenetic relationships between groups. Migration edges are subsequently added between branches with varying strength (branch weight = *w*) and directions to determine whether incorporating admixture events improves the likelihood of the tree given the genetic data. *Mytilus edulis* was used as an outgroup to focus inferences on recent admixture events and we accounted for linkage disequilibrium by performing analyses on windows of 100 variants. We examined the residual plot of pairwise population genetic covariances to infer the possibility of gene flow, where negative residual standard error values suggest closer relationships between populations than compared to the population tree with no migration events. We then modelled 1–10 migration events sequentially to see whether adding migration edges to the phylogeny improved the likelihood fit to the data. We calculated the standard error of migration events with the –se option without sample size correction (option –noss). We used a stepwise comparison Akaike information criterion (AIC) between sequential migration models to determine whether adding a migration edge significantly improved the likelihood of the population tree. We calculated AIC values as (−2(ln(likelihood)) + 2K), where *K* is the number of free parameters in the model. We did not consider additional migration events when the difference between nested models was less than two (∆AIC < 2).

Third, the three‐population (*f_3_*) test of admixture (Keinan, Mullikin, Patterson, & Reich, 2007; Reich, Thangaraj, Patterson, Price, & Singh, 2009) was used to verify evidence of migration inferred by TreeMix. We estimated the *f_3_* statistic using the *threepop* function. The *f_3_* statistic estimates whether allele frequency differences between each population combination deviate more than expected due to incomplete lineage sorting, thereby suggesting recent admixture. Significant migration is inferred if the *f_3_* statistic is negative and has a *z*‐score of ≤−3.8 (equivalent to a *p*‐value < .0001), which is determined through a jackknifing procedure over 100‐SNP windows.

### 2.4 Genomic analysis of species relationships

To visualize genomic relationships between Australian lineages against four northern *Mytilus* outgroup taxa (Table 1), we performed a genomic network analysis of individual haplotype sequences using the neighbour‐net method in SplitsTree4 v4.14.6 (Huson & Bryant, 2006). The phylogenetic network was generated from a concatenated nucleotide sequence (constructed in R) consisting of 27,343 SNPS from 2,620 nuclear contigs, using default settings. We also estimated a phylogenetic network based on 144 SNPs from 12 protein‐coding female mitochondrial genes. The distinctive *Mytilus* male mitotypes were not recovered due to low coverage in the transcriptome data.

To quantify how species relationships between *M. planulatus* and northern hemisphere *Mytilus* species vary across the nuclear genome, we estimated the relative contributions of three possible topologies (i.e. grouping *M. planulatus* with one of three outgroup species: *M. galloprovincialis* (Mediterranean)*, M. edulis* or *M. trossulus*) to the nuclear species tree using a heuristic topology weighting analysis in TWISST (topology weighting by iterative sampling of subtrees; Martin & Van Belleghem, 2017). TWISST estimates the weight or contribution of all possible unrooted topologies for each locus by resampling a single haplotype per taxon to generate all possible subtrees for that locus. To minimize unresolved topologies due to present day introgression, we included only putative non‐introgressed *M. planulatus* samples from Tasmania showing no evidence of admixture in initial analyses (discussed in section above). We also excluded the most distant outgroup, *M. californianus,* to limit comparisons to three possible topologies. Consensus haplotypes for each locus were analysed individually; we inferred locus‐specific genealogies with the R package ‘Ape’ using the neighbour‐joining method and F84 distances (Felsenstein & Churchill, 1996). To exclude poorly resolved phylogenies (e.g. Martin & Van Belleghem, 2017), we performed the analysis on a subset of 343 genealogies with a minimum tree length of 0.025, which is equivalent to 5 SNPs every 200 bp.

### 2.5 Approximate Bayesian Computations (ABC) of demographic history

**Figure 1:**
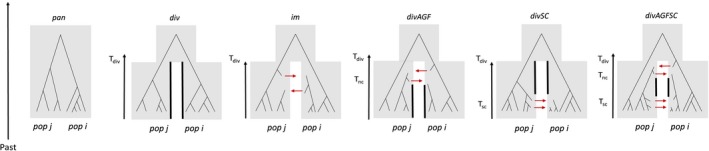
Competing divergence models between northern *M. galloprovincialis* (*pop j*) and native *M. planulatus* (*pop i*). Models assumed constant effective population sizes and divergence from an ancestral panmictic population at time *T_d_*_iv_. The *pan* model assumes populations belong to the same gene pool. The *div* model assumes populations evolve independently with no gene flow since their divergence. In the *im* model, populations diverge with ongoing gene flow to the present day. The *divAGF* model assumes bidirectional migration is restricted to the early stages of speciation from *T*_div_ to a more recent time (*T*_nc_) up to the last glacial maximum (20,000 years ago), after which populations evolve independently with no migration. This scenario is consistent with transequatorial migration between hemispheres facilitated by cyclical glacial cooling of the oceans during the late Pleistocene. In the *divSC* model, populations evolve in allopatry until recent human‐mediated secondary contact (*T*_sc_), when populations begin to exchange genes. This scenario tests explicitly for the presence of post‐introduction gene flow from northern *M. galloprovincialis* into Australian populations assuming that the onset of migration occurs after the earliest record of European contact (<600 years ago). Finally, the *divAGFSC* model assumes that populations diverged with ancient migration for a period of time, after which they evolve in allopatry; genetic exchange is re‐established at *T*_sc_ following recent secondary contact via human‐mediated introductions

We used an ABC framework to test the hypothesis that *M. planulatus* from Tasmania (putative endemic lineage) have experienced an independent evolutionary history from northern *M. galloprovincialis*. We evaluated six alternative models of Australian *Mytilus* origins representing a spectrum of divergence histories between northern and southern taxa: panmixia (*pan*), divergence in isolation (*div*), isolation with migration (*im*), divergence with ancient gene flow (*divAGF*), divergence with recent (invasive) secondary contact (*divSC*) and divergence with ancient gene flow and recent secondary contact (*divAGFSC*) (Refer to Figure 1).

We compared the genomic backgrounds of *M. planulatus* sampled in Tasmania against two divergent *M. galloprovincialis* lineages from the Mediterranean and Atlantic. Because of the power afforded by analysing large numbers of loci across the genome (i.e. thousands of independent genealogies), model‐based inferences of isolation and migration are robust to small sample sizes (i.e. *n* = 5–10) (Fraïsse, Roux, et al., 2018; Robinson, Bunnefeld, Hearn, Stone, & Hickerson, 2014). Details relating to (a) the filtering of empirical genetic data sets, (b) parameterizing and generating coalescent simulations of genetic data under separate demographic models, (c) demographic model selection, (d) model validation, (e) incorporating parameter heterogeneity and (f) demographic parameter estimation are outlined in the Appendix S1.

Briefly, as input for ABC analyses, we mapped reads against a reduced protein‐coding *M. galloprovincialis* transcriptome assembly and called variants using the *reads2snps* program. Subsequent filtering and analyses were conducted using custom R scripts (https://github.com/dinmatias) implementing an existing ABC pipeline (Roux et al., 2016; https://github.com/popgenomics/popPhylABC). The resulting empirical data sets consisted of 1,362 loci (Mediterranean‐Tasmania) and 1,539 loci (Atlantic‐Tasmania). For the simulated data, we used *msnsam* to generate one million multilocus simulations under each demographic model, for each population pair (Figure 1; Ross‐Ibarra et al., 2008). Initial models assumed equal (homogeneous) effective population size (*N*_e_) among loci and homogeneous migration rates (*m*) every generation. Each simulation was parameterized by model‐specific demographic parameters (Table S1) that are described in the Appendix S1. A standard set of 39 summary statistics (e.g. Fraïsse, Roux, Welch, & Bierne, 2014) of divergence and polymorphism were calculated for each simulation and for the empirical genetic data using *mscalc* (Ross‐Ibarra et al., 2008).

We evaluated the posterior support for alternative demographic models by performing a categorical regression (neural network method) on the model identity and summary statistics of the posterior samples (Beaumont, 2010) using the packages ‘abc’ (Csilléry, François, & Blum, 2012) and ‘nnet’ (Ripley, Venables, & Ripley, 2016) in R. We validated the power of our approach by performing the same analyses on 1,000 pseudo‐observed data sets (PODS) for each model simulated from the prior distribution. From this cross‐validation, we determined the overall precision (rate of correctly supporting a true model) and misclassification (type I error: rate by which incorrect models are supported) of our approach.

For initial ABC comparisons, simulated demographic models assumed genome‐wide homogeneous *N*_e_ and *m*. However, modelling the effects of linked selection on genome‐wide variation has been shown to improve the accuracy of demographic inferences in *Mytilus* species (Roux et al., 2014) and other semi‐isolated marine taxa (e.g. *Ciona sp.,* Roux et al., 2013; sea bass, Tine et al., 2014; *Salmo salar*, Rougemont & Bernatchez, 2018). To account for the combined effects of variable among‐locus rates of genetic drift and differential migration (Roux et al., 2016), we re‐simulated a series of nested models incorporating heterogeneous *N*_e_ and/or heterogeneous *m* under the best demographic scenario (inferred from initial homogeneous model comparisons) to estimate demographic parameters. We varied the initial *N*_e_ and *m* values for a certain proportion of loci by either (a) decreasing the initial parameter value (hetero1) or (b) allowing loci to have a lower or higher parameter values than the initial draw (hetero2). Finally, demographic parameters were estimated for each population pair using the posterior distribution approximated by accepted simulations under the best inferred demographic model.

## 3 RESULTS

### 3.1 Population structure and evidence for genome‐wide admixture

**Figure 2:**
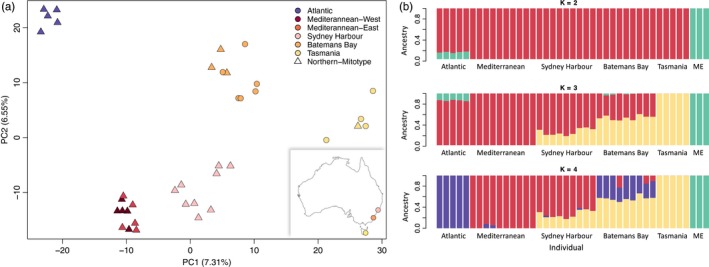
(a) Principal component analysis of three populations sampled in Australia (geographic location shown in inset map) and northern *M. galloprovincialis* from its native range in the Atlantic and Mediterranean Sea. Colours correspond to populations, and individuals marked with a triangle indicate samples carrying northern clade (*M. galloprovincialis)* mitochondrion (refer to Figure 4). (b) ADMIXTURE analyses for *K* = 2–4 genetic clusters, including *M. edulis* (ME) as an outgroup taxon. Each bar represents an individual with genetic elements belonging to one or more ancestral clusters, corresponding to different colours

Principal component analysis of 20,509 SNPs revealed separation between Australian samples and northern *M. galloprovincialis*, explaining 7.31% (PC1) and 6.55% (PC2) of variance among individual genotypes (Figure 2a). Populations from Sydney Harbour and Batemans Bay showed intermediate placement between samples from Tasmania and northern *M. galloprovincialis* populations. Divergence of these populations across the second PC axis points to likely admixture with divergent lineages of *M. galloprovincialis* from the Mediterranean and Atlantic. ADMIXTURE analyses for *K* = 4 clusters discriminated between genetic groups identified in the PCA (Figure 2b). Individuals sampled in Batemans Bay and Sydney Harbour showed greater than 50% shared ancestry proportions with northern *M. galloprovincialis*, suggesting at least two independent introductions into Australia of divergent source lineages: Atlantic *M. galloprovincialis* into Batemans Bay and Mediterranean *M. galloprovincialis* into Sydney Harbour, with subsequent admixture with these native populations. Analyses for all K clusters did not provide evidence of mixed ancestry proportions in Tasmania. Admixture proportions were consistent when analyses were performed using one SNP per contig to account for linkage effects, as nearby SNPs are not independent (Figure S2).

**Figure 3:**
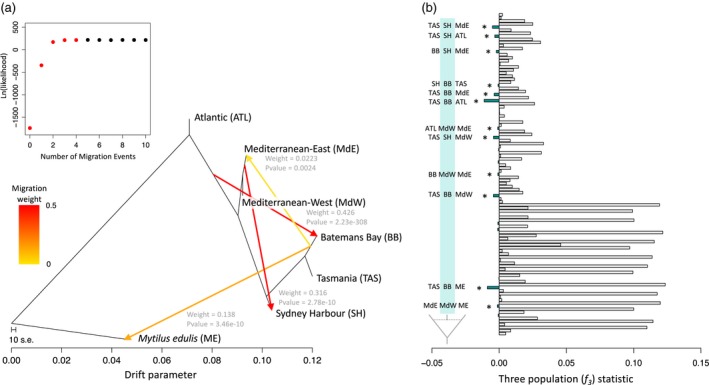
Tests of migration inferred by TreeMix. (a) Maximum‐likelihood population tree with four migration events including Australian *M. planulatus, M. galloprovincialis* and *M. edulis* as an outgroup. The addition of four admixture events significantly improved the fit of the population tree to the genetic data compared to a model with no migration. (b) The right panel shows *f*_3_ statistics for all population combinations including *M. edulis* as an outgroup. Significant and negative *f*_3_ values indicated with an asterisk and the corresponding three‐population combination is shown to the left of the asterisk, where the middle population marked with green shows evidence of admixture with putative ancestral populations indicated on either side

For TreeMix analyses, the population tree without migration explained 95.88% of variation in the allele frequency covariance matrix based on 34,097 SNPs (Figure S3). However, we observed high residual covariance between both Sydney Harbour and Batemans Bay with northern populations. The addition of four admixture events explained more than 99% of the genotypic variance and provided the highest likelihood fit (based on stepwise AIC comparisons) compared to models with fewer migration edges (Figure 3a); however, only two out of the four migration edges (those into Australian populations) accounted for most of the explained genotypic variance. We found significant *p*‐values (*p *« .001) for individual migration edges, although the direction of the migration edges should not be interpreted at face value (Figure 3a).

Results from TreeMix confirmed evidence for introgression from eastern Mediterranean *M. galloprovincialis* into Sydney Harbour (*w* = 33%) and migration from the Atlantic *M. galloprovincialis* population into Batemans Bay (*w* = 40%), suggesting contemporary admixture between native and introduced populations. Results did not provide evidence for migration into Tasmanian mussels. The slight signal of admixture between Batemans Bay and *M. edulis* suggests shared genetic elements are likely a result of secondary contact with Atlantic *M. galloprovincialis* populations that share ancestry with *M. edulis* through past and ongoing introgression (Fraïsse et al., 2016). The weak strength of this admixture event (*w* = 12%) is consistent with small proportions of *M. edulis* ancestry observed among Atlantic *M. galloprovincialis* individuals in clustering analyses (Figure 2b; *K* = 3). Similarly, slight evidence of admixture between the eastern Mediterranean and Batemans Bay likely indicates allele sharing with Mediterranean *M. galloprovincialis* through introgression with Atlantic populations prior to introduction; however, this migration edge was not strongly supported (*p *> .001; Figure 3a).

For *f_3_* statistics, we found significantly negative values (*p* < .0001) for almost all population combinations involving either Sydney Harbour or Batemans Bay as the admixed population, with *M. planulatus* (Tasmania) and northern *M. galloprovincialis* as putative ancestral populations (Figure 3b). Additionally, we detected signatures of *M. edulis* genetic elements in both Australian (e.g. Batemans Bay) and Mediterranean *M. galloprovincialis* (Mediterranean‐West) genetic backgrounds. Three‐population tests involving Tasmania as an admixed population did not yield significant values for any population combinations, supporting the hypothesis that samples in this region are representative of the endemic lineage.

### 3.2 Genomic analysis of species relationships

**Figure 4:**
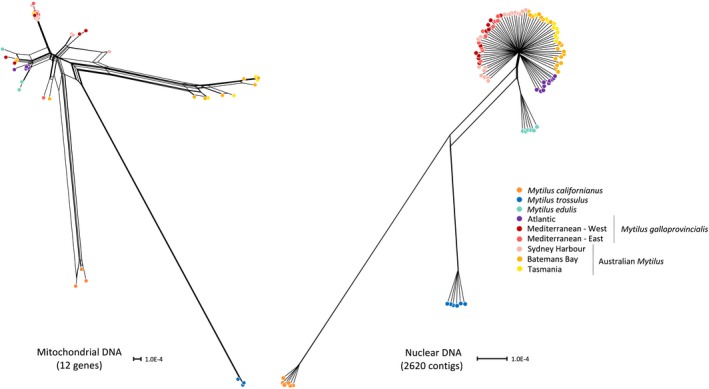
Consensus haplotype genetic network of Australian samples (*M. planulatus* samples from Tasmania are shown in yellow) and four outgroup taxa. Network phylogenies are constructed from 12 mitochondrial genes (left) and 2,620 nuclear contigs (right)

Consensus haplotype genetic networks of Australian samples and four *Mytilus* outgroup taxa constructed from 12 mitochondrial genes and 2,620 nuclear contigs revealed discordance between the mitochondrial tree and the average nuclear tree. Individuals carrying the Australian (female) mitotype formed a distinct divergent clade (Figure 4). In contrast, the same individuals clustered together alongside *M. galloprovincialis* when nuclear loci were analysed. TWISST analyses of species relationships corroborated that gene trees grouping *M. planulatus* with *M. galloprovincialis* dominated the nuclear genome (Figure S4). The mean weighting for topologies placing *M. planulatus* as a sister species to the invasive taxon was 54%, supporting a close relationship between these two species (Figure S4). Only 39/343 loci (11%) had fully resolved topologies (topology weight = 1.0), all of which grouped *M. planulatus* with *M. galloprovincialis.* Visual inspection of these topologies revealed that all loci are paraphyletic (do not form species‐specific clades) with *M. galloprovincialis,* suggesting high levels of ongoing incomplete lineage sorting in Tasmanian samples or genetic exchange, although we did not recover evidence supporting introgression in this population in any analyses. Topologies grouping *M. planulatus* with *M. edulis* and *M. trossulus* showed mean weightings across contigs of 21% and 23%, respectively (Figure S4), corroborating ancient incomplete lineage sorting resolved into multifarious gene tree topologies in the *Mytilus* species tree phylogeny.

### 3.3 Historical demographic inference using ABC

#### 3.3.1 Levels of endemic genetic diversity and divergence at synonymous sites

The majority of biallelic polymorphic sites were shared between *M. planulatus* and *M. galloprovincialis* (average shared polymorphic sites across loci = 5.36–5.83), compared to private polymorphisms in *M. planulatus* (average private polymorphic sites = 2.87–4.07). Levels of nucleotide diversity at synonymous sites (averaged across loci) were similar for all populations based on Tajima's pi (Tajima, 1983) ranging between 0.023 and 0.024 for Tasmanian *M. planulatus* and 0.021 (Mediterranean) and 0.026 (Atlantic) for northern *M. galloprovincialis*. Pairwise comparisons indicated low population differentiation evidenced by few fixed variants (0–0.02 averaged across contigs) and low *F*_ST_ or absolute (*d*_xy_) and net (*D*_a_) divergence values between Tasmania and Mediterranean (mean *F*_ST_ = 0.052; *d*_xy_ = 0.25; *D*_a_ = 0.003) or Atlantic (mean *F*_ST_ = 0.087; *d*_xy_ = 0.30; *D*_a_ = 0.005) *M. galloprovincialis*, indicating that population differentiation is largely driven by the presence of private alleles. Departures of the site frequency spectrum measured as mean Tajima's *D* values (Tajima, 1989) varied between populations; we observed negative values close to neutrality in Tasmania (average *D *= −0.151) and greater negative values in *M. galloprovincialis* (Mediterranean *D *= −0.475; Atlantic *D *= −0.445), indicating an excess of rare alleles due to population expansions, gene flow from unsampled populations or signatures of directional selective processes.

#### 3.3.2 Historical isolation between northern and southern hemispheres

We compared six models of divergence between Tasmanian *M. planulatus* and *M. galloprovincialis* using an ABC framework. We first inferred the best demographic model by comparing models with homogeneous among‐locus parameters. The model of divergence with ancient gene flow (*divAGF*) received the highest posterior support (>82% posterior probability using neural network inference and acceptance threshold 0.001) for both population pairs (Table 2). The strict divergence model (*div*) provided the second highest posterior probability in most comparisons. We observed consistent rejection of the isolation‐with‐migration (*im*) model suggesting that historical gene flow was followed by divergence in isolation. Model comparisons returned no support for panmixia and weak support for all models that included recent gene flow associated with contemporary introductions < 600 years ago.

**Table 2:** Summary of demographic model selection under an approximate Bayesian computation (ABC) framework

Tolerance	Method	Demographic model probability: proportion of accepted simulations					
		*pan*	*Div*	*im*	*divSC*	*divAGF*	*divAGFSC*
(A)							
0.001	Rejection	0.0000	0.1320	0.0417	0.0188	**0.7835**	0.0240
0.01	Rejection	0.0000	0.1955	0.0708	0.0546	**0.6130**	0.0660
0.001	Rejection	–	0.1588	0.0266	0.0100	**0.7894**	0.0152
0.01	Rejection	–	0.1972	0.0664	0.0398	**0.6421**	0.0545
0.001	Neural Net	–	0.0328	0.0375	0.0056	**0.9094**	0.0142
0.01	Neural Net	–	0.0618	0.0051	0.0026	**0.9267**	0.0038
(B)							
0.001	Rejection	0.0000	0.3868	0.0037	0.0015	**0.6047**	0.0033
0.01	Rejection	0.0000	0.3052	0.0135	0.0069	**0.6654**	0.0090
0.001	Rejection	–	0.4252	0.0036	0.0028	**0.5654**	0.0030
0.01	Rejection	–	0.3129	0.0116	0.0062	**0.6611**	0.0081
0.001	Neural Net	–	0.0527	0.0019	0.0059	**0.8273**	0.1123
0.01	Neural Net	–	0.0408	0.0010	0.0009	**0.9557**	0.0015

Note

Model posterior probabilities assuming homogeneous *N*_e_ and *m* parameters for A) the Mediterranean‐Tasmania population pair; and B) the Atlantic‐Tasmania population pair. In model comparisons where not all six demographic models had accepted values within the applied threshold, the simple rejection method (i.e. linear regression) was applied. Bold indicates the highest probability model for each comparison.

Model choice validation indicated that we could discriminate between the best inferred model (*divAGF*) and models including recent genetic exchange (*im, divSC, divAGFSC*). The *divAGF* model had the highest posterior probability in 59% (Atlantic) and 61% (Mediterranean) of model comparisons using PODs generated under the same model (i.e. precision; Figure S5, Table S3). However, we found that 41% (Atlantic) and 39% (Mediterranean) of PODs simulated under the *divAGF* model were misclassified as divergence in isolation (*div)*. Measures of robustness in the accuracy of model discrimination indicated a minimum threshold for model probability ≥ 83% required to yield a robustness of 95% or greater for the *divAGF* model, corroborating initial model choice inference (Table 2). Overall, there was clear discrimination between models excluding (*divAGF, div*) and including (*im, divSC, divAGFSC*) ongoing gene flow since divergence, suggesting that *M. planulatus* and *M. galloprovincialis* have experienced a period of historical isolation.

#### 3.3.3 Genome‐wide heterogeneous genetic drift and migration

Comparisons of the heterogeneous models (under the best demographic scenario; *divAGF*) allowing among‐locus variation in *N*_e_ and *m* provided an improved model fit to the observed genetic data when compared to the *divAGF* model with homogeneous parameters (Table S4). Specifically, we found that models allowing *N*_e_ to vary both below and above the initial *N*_e _parameter value (hetero2) and where *m* was either homogeneous (Mediterranean‐Tasmania) or heterogeneous (Atlantic‐Tasmania) outperformed other models (*N*_e_ homo and *N*_e_ hetero1) with a cumulative posterior probability > 90% for both population comparisons. Substantial support for hetero2 models suggests that heterogeneity in *N*_e_ is better captured with models incorporating both extremes of variation across loci; processes leading to both low levels of variation (e.g. due to linked selection, Rougemont & Bernatchez, 2018) and high diversity at synonymous loci (e.g. due to polygenic balancing selection, Charlesworth, 2006; introgression from unsampled ghost populations, Butlin et al., 2014; variable levels of ancestral diversity, Guerrero & Hahn, 2017) are therefore important factors to account for when reconstructing population histories and estimating demographic parameters.

#### 3.3.4 Demographic parameter inference from the best models

**Figure 5:**
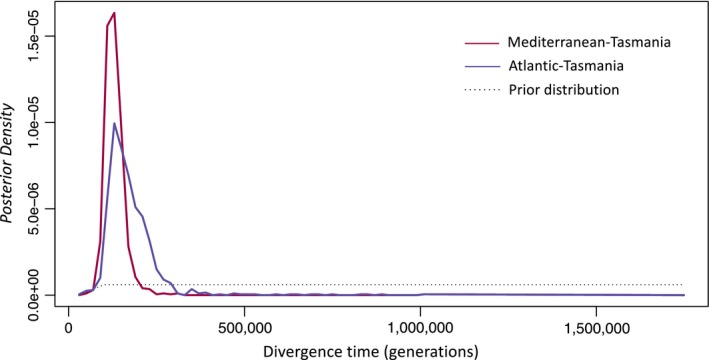
Posterior distributions for divergence time (*T*_div_) parameter estimates inferred from ABC analyses for two population pairs based on highest probability demographic model (*divAGF*) accounting for heterogeneous *N*_e_ and *m* parameters. Parameter plots correspond to models with the highest estimated probability in which the *N*_e _parameter was hetero2 and *m* was either homogeneous (Mediterranean‐Tasmania; mode: 116,352 generations) or hetero1 (Atlantic‐Tasmanian; mode: 134,157 generations)

For the best heterogeneous *divAGF* models, divergence time parameters were contained within the prior distribution and well differentiated in both population comparisons (Mediterranean‐Tasmania Mode: 116,352 generations, CI 95% [90,858–173,929]; Atlantic‐Tasmania Mode: 134,157 generations, CI 95% [98,371–274,804]; Figure 5). Divergence time estimates suggest that Australian *M. planulatus* started diverging in allopatry between 100,000 and up to 600,000 years ago and are likely to have experienced low levels of historical gene flow. However, estimates of ancient migration (*ma*), including the time of onset of ancient gene flow since the present (*T*_nc_), were poorly resolved and could not be estimated with accuracy.

## 4 DISCUSSION

Detecting species introductions and documenting cases of recent introgression are notoriously challenging when native and introduced species inhabit the “grey zone” of the speciation continuum, where species boundaries are often contentious (Roux et al., 2016). Here, we utilize the power of transcriptome‐wide markers to uncover the complex divergence history of *Mytilus* mussels in Australia. Unexpectedly, we find that contemporary introductions of *M. galloprovincialis* into south‐eastern Australia originate from genetically distinct northern hemisphere lineages, implicating at least two independent introductions into this region. Contingent on resolving species relationships, we also provide evidence that both introductions are associated with recent admixture with the native Australian taxon, *M. planulatus*. Through increased genome‐wide resolution of species relationships, combined with demographic modelling, we validate that *M.* *planulatus* sampled in Tasmania are representative of the endemic Australian lineage and have been isolated from northern *M. galloprovincialis* for at least 100,000 years. Taken together, our study demonstrates the utility of genomic data for detangling the contributions of contemporary invasive hybridization from signatures left by historical gene flow and recent divergence histories in high dispersal marine species.

### 4.1 Multiple introductions from northern hemisphere source lineages

Our study uncovers two new and important results regarding *M. galloprovincialis* introductions and its interactions with endemic southern hemisphere taxa, building upon previous genetic studies (e.g. Borsa et al., 2007; Gérard et al., 2008; Westfall & Gardner, 2010). First, we demonstrate that introductions into Australia are derived from genetically distinct source lineages of *M. galloprovincialis* from the Mediterranean Sea and the Atlantic coast of Europe. Genotypic variance across nuclear SNPs revealed differentiation between populations from Sydney Harbour and Batemans Bay from a more divergent Tasmanian lineage and *M. galloprovincialis* sampled from its native range (Figure 2a). Within these two populations, all individuals displayed mixed ancestry with high genomic contributions (i.e. 33%–82% ancestry proportions; Figure 2b) from northern *M. galloprovincialis*, pointing to at least two contemporary introductions of divergent northern genotypes into mainland Australia (i.e. Westfall & Gardner, 2010). The second important finding is that both introductions have led to repeated instances of hybridization and introgression with native *M. planulatus*. Tests of migration in TreeMix revealed strong evidence for introgression from eastern Mediterranean *M. galloprovincialis* into Sydney Harbour and from Atlantic *M. galloprovincialis* into Batemans Bay (Figure 3a). Aside from the Tasmanian population, a striking observation is that we did not identify any nonintrogressed *M. planulatus* or pure northern *M. galloprovincialis* individuals among Australian samples. This result suggests that admixture may be widespread and that both introductions are accompanied by introgression, most likely from the native into the introduced genetic backgrounds (Currat, Ruedi, Petit, & Excoffier, 2008).

Multiple introductions are a common feature of biological invasions for many non‐native marine species (Riquet, Daguin Thiébaut, Ballenghien, Bierne, & Viard, 2013; Rius et al., 2015; Viard et al., 2016). Successive introductions of large numbers of larvae are likely to promote secondary contact and subsequent admixture between genetically differentiated lineages (Keller & Taylor, 2010; Rius & Darling, 2014). For example, differentiated lineages of the invasive European green crab, *Carcinus maenas,* have been independently introduced into eastern Northern America (Darling, Bagley, Roman, Tepolt, & Geller, 2008; Tepolt & Palumbi, 2015), and postintroduction admixture between warm‐adapted and cold‐adapted lineages has been proposed as a factor in the establishment of invasive genotypes beyond their previous range limits (Darling, Tsai, Blakeslee, & Roman, 2014; Jeffery et al., 2018; Tepolt & Somero, 2014). Our findings are in line with general perceptions that successful marine introductions are likely to involve propagules from multiple and potentially diverse sources (Lockwood, Cassey, & Blackburn, 2005; Rius et al., 2015). It is tempting to speculate that variation in thermal physiology has contributed to successful introductions of Atlantic *M. galloprovincialis* in more southern (cooler) habitats relative to Sydney Harbour, where Mediterranean *M. galloprovincialis* predominate. However, there is no evidence to date suggesting differences in temperature tolerance among *M. edulis*‐introgressed Atlantic mussels and those of purely Mediterranean origins. Similarly, whether post‐introduction admixture between divergent *M. galloprovincialis* lineages has enhanced the success of introduced populations will require additional investigations examining these synergies (Rius et al., 2015).

While genetic studies in marine systems have not explicitly investigated whether introgression directly promotes successful introductions, it is evident that hybridization can influence both invasive and native species in a number of ways (Le Roux & Wieczorek, 2009; Sloop, Ayres, & Strong, 2009). Hybridization may alleviate Allee effects on introduced populations through purely demographic processes (Gagnaire et al., 2018; Johannesson, 1988; Mesgaran et al., 2016). Differential introgression of introduced diversity into native genomic backgrounds may also promote the spread on non‐native alleles and increase opportunities for adaptation in introduced environments (Fitzpatrick et al., 2010). In Australia, we find signatures of divergent *M. edulis* genetic elements in one admixed population (i.e. Batemans Bay) likely obtained through post‐introduction admixture with introgressed Atlantic *M. galloprovincialis* (rather than direct gene flow with *M. edulis*). These findings are consistent with previous investigations implicating asymmetric introgression of *M. edulis* genes into Atlantic *M. galloprovincialis* populations in south‐western Europe (Bierne, Borsa, et al., 2003; Fraïsse et al., 2014; Gosset & Bierne, 2013; Rawson & Hilbish, 1998; Roux et al., 2014). Additionally, a recent study (Simon et al., 2019) has shown that Mediterranean‐lineage *M. galloprovincialis* introduced into five Atlantic shipping ports display mixed *M. edulis* ancestry and genetic separation from local genomic backgrounds, suggesting that admixture with *M. edulis* has occurred prior to regional introductions. While it is not evident whether introgressed *M. edulis* genetic elements are adaptive for introduced populations, similar patterns of introgression in Atlantic ports (Simon et al., 2019) and Australian introduced populations (this study) raise important considerations regarding the possibility of differential introgression of divergent outgroup variants into native genetic diversity, where continued dispersal of admixed genotypes may benefit colonizers (Keller & Taylor, 2010; Rius & Darling, 2014). Given the small numbers of sampled individuals in the present study, however, and the potential for high variance in allele frequencies, we could not test specific hypotheses regarding levels of gene flow at specific loci to explore these conjectures. Nevertheless, our findings reinforce the notion that sufficient sampling of closely related sister lineages in both sympatric (i.e. endemic *M. planulatus*) and allopatric ranges (i.e. *M. edulis* outgroup) is imperative for accurate interpretations of intraspecific genomic differentiation and signatures of introgression associated with marine introductions.

### 4.2 Late‐Pleistocene divergence between native and introduced Australian mussels

Documenting the spread of *M. galloprovincialis* in Australia has been the subject of a number of previous genetic investigations; however, distinguishing between introduced and native taxa has been hampered by low genetic differentiation between populations and high levels of marker discordance (Westfall & Gardner, 2010). Representing a much larger proportion of genetic variation than previous approaches, we confirm strong species tree discordance between mitochondrial and genome‐wide nuclear loci (Gérard et al., 2008; Hilbish et al., 2000). Network analysis of 12 protein‐coding mitochondrial genes from Australian populations placed southern lineage mitochondrial haplotypes in a clade divergent from northern *M. galloprovincialis* (Figure 4). Additionally, haplotypes across all genes remained paraphyletic for *M. galloprovincialis* and *M. edulis* sister taxa despite 2.5 million years of divergence (Roux et al., 2014), consistent with historical isolation between southern and northern hemisphere lineages that predates the split between *M. edulis* and *M. galloprovincialis* (Gérard et al., 2008). In contrast, variation across the nuclear genomic background of Australian mussels alongside northern hemisphere taxa validated strong genetic affinities to *M. galloprovincialis*, suggesting a closer genetic relationship to the invasive taxon than implicated by mitochondrial loci (e.g. Fraïsse, Haguenauer, et al., 2018; Hilbish et al., 2000).

Discordant species relationships between mitochondrial and nuclear genomes are expected to arise due to the fourfold difference in effective population size between genomes, with stronger genetic drift and faster lineage sorting in mitochondrial genes (Toews & Brelsford, 2012). For example, strong differentiation in the mitochondrial genome of *M. planulatus* may be explained by historical bottlenecks associated with the first colonizations of Australia by northern *Mytilus *taxa followed by a period of allopatry (Gérard et al., 2008). Introgression through past or contemporary gene flow may erode differentiation at nuclear loci, further pronouncing mito‐nuclear discordance between northern and southern hemisphere lineages. TreeMix and ABC inferences, however, recovered weak support for contemporary gene flow from northern *M. galloprovincialis* into Tasmanian mussels (Figure 3), suggesting that samples in this region are largely representative of the endemic Australian taxon. We also found little evidence that Tasmanian mussels are introgressed with *M. edulis* through past admixture as suggested by previous authors (e.g. Borsa et al., 2007). Instead, high variance in gene tree topologies in TWISST indicated extensive levels of incomplete lineage sorting with northern *M. edulis* and *M. trossulus*, which may account for the presence of outgroup alleles in some populations (Figure S4; Westfall & Gardner, 2013). Furthermore, paraphyly among *M. planulatus* and *M. galloprovincialis* haplotypes in locus‐specific topologies (analysed in TWISST) suggests ongoing incomplete lineage sorting (i.e. shared ancestral polymorphism) between native and introduced taxa.

Focusing on Tasmanian mussels as representative of endemic *M. planulatus*, demographic inferences provided the strongest support for a model of divergence with low levels of historical gene flow with northern *M. galloprovincialis*. Parameter estimations indicated recent divergence times between 100,000–600,000 years ago, suggesting that *M. planulatus* likely differentiated from proto‐*M. galloprovincialis* postdating the separation of northern hemisphere species. While divergence time estimates assume a generation time of 2 years, these approximate values are compatible with midden fossils placing endemic *Mytilus* in Australia since the end of the late glacial retreat (>10,000 years bp). Estimates are also consistent with previous studies proposing separation times between northern and southern hemisphere *Mytilus* species ~0.5 to 1.3 million years ago based on mitochondrial loci (Gérard et al., 2008; Hilbish et al., 2000). More broadly, our findings support a scenario of parallel transequatorial migrations leading to the origins of southern hemisphere mussels, that is *M. planulatus* in Australasia which is more related to *M. galloprovincialis* (this study) and *M. platensis* in South America and the Kerguelen Islands which is more related to *M. edulis* in the northern hemisphere (Fraïsse, Haguenauer, et al., 2018). Under this scenario, however, mitochondrial introgression swamping following secondary contact between *M. galloprovincialis* and *M. edulis* (Fraïsse, Haguenauer, et al., 2018; Gérard et al., 2008) has been proposed to reconcile incongruent patterns of deep divergence at mitochondrial loci and reciprocal monophyly (with northern haplotypes) for all southern hemisphere lineages (Gérard et al., 2008).

Based on the genomic data presented here, we infer that shared polymorphisms and modest signals of genome‐wide differentiation between *M. planulatus* (Tasmania) and *M. galloprovincialis* are mostly due to recent divergence histories possibly associated with historical gene flow (likely facilitated by glacial melting and the formation of cool‐water refugia across the equator; Lindberg, 1991). However, we found no evidence for contemporary introgression from human‐mediated introductions of *M. galloprovincialis* in this region, suggesting that Tasmanian mussels represent naturally occurring endemic genetic diversity. *Mytilus planulatus* is currently recognized as valid nomenclature in the World Register of Marine Species (WORMS) database. Strong sequence similarities and permeable barriers to gene flow with *M. galloprovincialis,* however, support previous proposals to assign the endemic Australian lineage with a regional subspecies status (e.g. Borsa et al., 2007; Daguin & Borsa, 2000; Gérard et al., 2008; Hilbish et al., 2000; Westfall & Gardner, 2010). Further clarification will be required on whether *M. planulatus* has experienced historical introgression with other southern clades in New Zealand and South America (e.g. Larraín et al., 2018). Genetic investigations to date have used either insufficient loci to resolve species relationships and migration (e.g. Westfall & Gardner, 2010) or have sampled only a single landmass (e.g. Gardner et al., 2016). A recent genetic survey has confirmed genomic differentiation between southern clades (Gérard et al., 2008), including the Kergulen islands; however, introgression with *M. planulatus* was not investigated (Fraïsse, Haguenauer, et al., 2018). The relationship between two southern *Mytilus* taxa (i.e. *M. planulatus* and *M. platensis*) will therefore require further investigation.

### 4.3 Monitoring M. galloprovincialis introductions in Australia and implications for invasive species research

*Mytilus* mussels are well‐known ecosystem engineers, and changes in their abundance and range could impose significant alterations to intertidal communities (Braby & Somero, 2006). Ecological impacts of *M. galloprovincialis* in other parts of the world, including niche displacement of native taxa (California, Rawson et al., 1999; South Africa, Bownes & McQuaid, 2006) and negative effects on aquaculture populations through parasite hitchhiking (Dias et al., 2014; Jones & Creeper, 2006), warrant caution against *M. galloprovincialis* introductions into Australian coastlines and industries. For the case of Australian *Mytilus*, however, it is evident that many markers are required for identification of introduced and endemic populations. Our results confirm that diagnostic methods used to identify hemisphere origins based on mitochondrial loci (e.g. Ab Rahim et al., 2016; Colgan & Middelfart, 2011; Dias et al., 2014) are not a reliable method for differentiating native and introduced *Mytilus* populations, especially when hybridization is pervasive. We also demonstrate that multiple introduced sources are only detected when significant structure exists in the native range. It is therefore likely that for many marine species, even genomic data will not be sufficiently robust to resolve exact sources of populations in the absence of strong differentiation, despite adequate sampling of native range variation (e.g. eastern versus western Mediterranean *M. galloprovincialis*). In such cases, surveys for high gene flow marine invasive species using environmental DNA (Kelly, Port, Yamahara, & Crowder, 2014) may not be able to detect intraspecific non‐native diversity or even interspecific variation, despite holding promise for identifying broader taxonomic groups (Bourne, Hudson, Holman, & Rius, 2018).

Knowledge regarding the potential ecological consequences associated with introduced populations will therefore be essential for monitoring and minimizing the spread of introduced lineages (Braby & Somero, 2006). Considering *Mytilus* as a case study, Saarman and Pogson (2015) recently documented low levels of asymmetric introgression from native *M. trossulus* into invasive *M. galloprovincialis* in its introduced range in California, where patterns of introgression were consistent with predictions that populations furthest from the source of introduction should experience stronger gene flow into the invading genomic background (Currat et al., 2008). These findings suggest that the direction of introgression and the potential for introgression swamping towards endemic taxa are likely driven by the relative sizes of native and non‐native populations, rather than by selection processes. Documented cases of introgression between invasive and native taxa, including newly described parallel hybrid zones between *M. planulatus* and *M. galloprovincialis,* provide opportunities to evaluate how introgression with native congeners may accelerate or impede the spread of introduced species. We hypothesize that an absence of pure parental individuals in the present study (with the exception of Tasmania) indicates significant contributions of introgression to the genomic composition of Australian mussels, likely during the early stages of the introductions. Future research in *Mytilus* and other marine invasive species should focus on temporal and spatial sampling of greater numbers of individuals to assess the rate and direction of introgression and the potential impacts of introgression swamping of native genomes on ecologically relevant timescales (Glotzbecker et al., 2016; Riley et al., 2003; Todesco et al., 2016). Such data will be critical for informing our general understanding of the role of hybrid zone expansions in marine invasive spread and the scope and potential of long‐term (evolutionary) impacts of biological introductions on receiving marine communities.

## CONFLICT OF INTEREST

None declared.

## Data Availability

The raw RNA‐seq data are deposited to the NCBI sequence read archive (BioProject ID: PRJNA560413). Genomic data sets that support the findings of this study are openly available in Genomic Observatories MetaDatabase (https://geome-db.org) and the Dryad digital repository at ://doi.org/10.5061/dryad.540cc05 (Popovic, Matias, Bierne, & Riginos, 2019a, 2019b, 2019c).
