# Supplementary material for: Twin introductions by independent invader mussel lineages are both associated with recent admixture with a native congener in Australia
Source: Evol Appl. 2019 Sep 18;13(3):515–32. doi: 10.1111/eva.12857 (PMC7045716; doi:10.1111/eva.12857)

Supplementary Material

Methods

RNAseq data processing and reference transcriptome assembly

We used Trimmomatic (v0.36) (Bolger et al. 2014) to remove adapter sequences from paired reads. Quality trimming was performed using a 4 bp sliding window, a phred-scale average quality score of 20 and a minimum size filter of 50 bp. We reduced redundancy among high-coverage reads (including rRNA contaminants) and discarded associated sequence errors by digitally normalising each dataset using Trinity's insilico_read_normalization.pl script with a default kmer size of 25 and maximum read coverage of 50 (Grabherr et al. 2011). Overlapping paired reads were merged using FLASH v1.2.11 (Magoč and Salzberg 2011) with a minimum overlap length of 10 bp. Three *M. galloprovincialis* individuals (those with the highest FLASH merging scores: the highest absolute number of reads merged into larger fragments) from three native-range populations (refer to Table 1) were used to make a reference transcriptome assembly intending to capture representative proportions of genetic variation in the *M. galloprovincialis* native range. These samples were used to create three population-specific *de novo* assemblies using Trinity v2.0.6 (Grabherr et al. 2011) with default parameters. The longest isoforms were extracted for each gene group for each assembly. The three reduced Trinity assemblies were meta-assembled using CAP3 (Huang and Madan 1999) with default parameters into a single high-quality *M. galloprovincialis* reference assembly.

We queried the resulting assembly against the Uniprot-Swissprot protein database using blastx with an e-value threshold of 10^{-3} for significant matches. Contigs with significant blast matches to likely environmental contaminants, including bacteria, fungi, viruses, protists (Alveolata), green (Viridiplantae) and red algae (Haptophyceae), and other eukaryotic contaminants (i.e. Euglenozoa) were removed using Biopython v1.68 and R (184,842 contigs; R Development Core Team 2017). Prior to genomic analyses, transcripts showing high sequence similarity in the reference assembly (i.e. likely derived from the same gene) were clustered using Cd-Hit-Est (Li and Godzik 2006; Fu et al. 2012) with a minimum sequence identity threshold of 95% of the shortest sequence. Finally, we removed transcripts with significant blastn matches (e-value 10^{-3}) to the *M. galloprovincialis* male (Genbank reference: FJ890850.1) and female (Genbank reference: FJ890849.1) mitochondrial genomes. The resulting 159,985 nuclear sequences were used as a reference assembly for variant discovery and as input for all downstream analyses.

Approximate Bayesian Computations (ABC) of demographic history

Empirical genetic data

We reduced the reference assembly to only contigs containing predicted open reading frames (ORFs), using Transdecoder (Haas et al. 2013); we identified protein-coding sequences greater than 100 amino acids and with significant matches to the Pfam protein database. This resulted in 52,364 transcripts with ORFs, including 16,151 complete protein-coding nuclear loci (in which both start and stop codons were detected). We mapped individual reads datasets (as described above) against this reduced complete protein-coding assembly and used the resulting BAM files as input for downstream ABC analyses. We conducted two pairwise population comparisons to calculate summary statistics: We compared the genomic backgrounds of *M. planulatus* sampled in Tasmania (putative endemic) with two divergent *M. galloprovincialis* lineages from its native range in the Mediterranean and Atlantic. For each dataset, the *reads2snps* program was used to predict individual genotypes based on a probabilistic maximum-likelihood framework. Genotypes below a minimum read depth of 10 and genotype posterior probability of 95% were removed as well as variants resulting from the misalignment of reads to paralogous contigs. Subsequent analyses were conducted on the output of *reads2snps* using custom scripts in R (available at https://github.com/dinmatias). The R scripts implement an existing pipeline available from the PopPhyl project (https://github.com/popgenomics/popPhylABC; Roux et al. 2016). For each pairwise population comparison, we used PolydNdS to retain only synonymous variants and transcripts above a minimum length of 30 synonymous sites. Following these filtering thresholds, we removed monomorphic loci and loci with missing haplotypes for any individuals. The resulting empirical datasets consisted of 1,362 loci for Mediterranean-Tasmania, and 1,539 loci for Atlantic-Tasmania population pairs.

Coalescent simulations of genetic data

We used msnsam, a modified version of the ms coalescent simulator, to generate one million multilocus simulations under each demographic model, for each population pair (Ross-Ibarra et al. 2008). Simulations assumed a neutral mutation rate μ=2.763x10^{-8} per bp per generation, which was scaled by the number of synonymous sites of each locus to obtain per-locus mutation rates. To account for recombination, we followed the recombination rate implemented by Roux et al. (2016), which is equal to 0.5 of the mutation rate. Initial models assumed equal (i.e. homogenous) effective population size (N_e) among loci and homogeneous migration rate, M=4 N_{ref} m every generation, where N_{ref} is the

reference effective population size and *m* is the proportion of each population consisting of new migrants each generation.

Each simulation was parametrised by model-specific demographic parameters randomly drawn from a uniform prior distribution (Table S1) generated by a modified version of the *priorgen* software (Ross-Ibarra et al. 2008). Effective population size parameters (N_i, N_j, and $N_{ancestral}$) were randomly drawn from a distribution of 1000-500,000 individuals. To inform the priors for time-related parameters, we capitalised on previous divergence estimates for *Mytilus* species translated to generations using a generation time of 2 years (e.g., Roux et al. 2014). Specifically, divergence time (T_{div}) was sampled from the interval 100,000-1,750,000 generations to capture the earliest estimated time of mitochondrial divergence between southern hemisphere taxa and *M. galloprovincialis* between 0.54-1.31 million years ago (Gérard et al. 2008). The upper bound for T_{div} was informed by the estimated splitting time between *M. trossulus* and the ancestor of *M. edulis* and *M. galloprovincialis* (~3.5 million years) that preceded potential periods of transequatorial migration associated with the late Pliocene about 3.1 million years ago (Lindberg 1991; Hilbish et al. 2000). The prior distribution for bidirectional ancient migration (*ma*) between northern and southern hemispheres was sampled between 0-0.0001 (equivalent to 0-200 migrants per generation when N_{ref} =500,000). We sampled the number of generations since ancient migration seized (T_{nc}) bounded by the interval T_{div}-10,000 generations (corresponding to the last glacial maximum). For the invasive migration parameter (*m*) we explored unidirectional gene flow values sampled on a broader interval *m*=0-0.5 into Tasmania. We sampled the onset of human mediated secondary contact (T_{sc}) on the interval 5-300 generations. A standard set of 39 summary statistics (e.g., Roux et al. 2014; Fraïsse et al. 2014) of divergence and polymorphism were calculated for each simulation and for the empirical genetic data using *Mscalc* (Ross-Ibarra et al. 2008).

Demographic model selection

To evaluate the posterior support for alternative demographic models, we obtained posterior samples from all simulated data by applying thresholds of 0.001 and 0.01. An acceptance threshold of 0.001 is equivalent to 6000 simulations that generated summary statistics falling closest to the observed empirical values (Blum and François 2009). To estimate the posterior probability of each model, we performed a categorical regression on the model indices and summary statistics of the posterior samples using the feed-forward neural network method (Beaumont 2010). Computations were performed with 50 trained neural networks and a maximum of 2000 iterations while weighing each posterior sample by an Epanechnikov kernel with a maximum value when the simulated values are

equal to the observed summary statistics. In comparisons where not all six demographic models had accepted values within the applied threshold, the simple rejection method (i.e. linear regression) was applied (Beaumont et al. 2010). All these procedures were conducted using the packages `abc` (Csilléry et al. 2012) and `nnet` (Ripley et al. 2016) in R.

To validate the ability to discriminate between alternative models, we simulated an additional 1000 pseudo-observed datasets (PODS) from the prior distribution for each demographic model to perform model checking. Using each POD as the new empirical dataset, we estimated posterior support for each model given the original simulated summary statistics utilising the model selection procedure (as described above). We then examined the rate by which our approach correctly supported the true model of the PODs (i.e. precision) and the rate of by which incorrect models are supported (i.e. misclassification or Type I error). From this validation procedure, we examined the minimum threshold for model probability that will give a robustness of 0.95 (Roux et al. 2016), that is, a 95% probability to correctly support a model given that its posterior probability is higher than the threshold. We applied this minimum probability to evaluate if the estimated posterior probability for the empirical data is robust.

Accounting for among-locus variation in genetic drift and migration

For initial ABC comparisons, simulated demographic models assumed genome-wide homogenous N_e and m. Accounting for differential rates of introgression and genomic variance in genetic drift that may result as an outcome of linked selection, however, has been shown to significantly improve the accuracy of demographic inferences in marine taxa (*Ciona sp.,* Roux et al. 2013; *Mytilus* species, Roux et al. 2014; sea bass, Tine et al. 2014; *Salmo salar*, Rougemont and Bernatchez 2018). To account for the combined effects of differential migration and variable among-locus rates of genetic drift, we re-simulated a series of nested models incorporating heterogeneous N_e and/or heterogeneous m under the best demographic scenario (inferred from initial homogeneous model comparisons) to estimate demographic parameters. Specifically, we were interested in whether these models provided an improved model probability (and parameter estimates) when compared to the best inferred model with homogeneous N_e and m.

In simulating these additional models, an initial value of N_e and m were randomly drawn as described above. These initial values of N_e and m were homogenously applied for all the loci (homo). To account for differential selection and migration across the genome, we varied the initial N_e and m values for a certain proportion of loci. This proportion was drawn from a uniform distribution [0-1]. The N_e and m of

these sets of loci were modified by (i) decreasing the initial parameter value (hetero1) or (ii) allowing the each of these loci to have a lower or higher parameter than the initial draw (hetero2). For each locus with modified N_e and m, the amount of decrease/increase was proportional to the initial values by a certain factor, which was sampled from a beta distribution with shape parameters drawn from the interval [1-50] for N_e and [1-20] for m. We simulated 1 x 10^6 simulations for a total of 13 additional heterogeneous models and calculated summary statistics as described above.

Demographic parameter inference

Demographic parameters were estimated for each population pair using the posterior distribution approximated by accepted simulations under the best demographic model. Parameter values were log transformed prior to regression to ensure that the posterior distribution was contained within the prior bounds (e.g., Estoup et al. 2004; Hamilton et al. 2005). We used 50 neural networks and a maximum of 2000 iterations to obtain weighted non-linear regressions of the parameters to the summary statistics from 1000 accepted simulations closest to the observed values (acceptance tolerance=0.001%). Parameter inference was based on estimated posterior means and 95% credible intervals when parameters were differentiated from the uniform prior.

Literature Cited

Beaumont, M. A. (2010). Approximate Bayesian computation in evolution and ecology. *Annual Review of Ecology, Evolution, and Systematics*, 41, 379–406.

Blum, M. G., & François, O. (2010). Non-linear regression models for Approximate Bayesian Computation. *Statistics and Computing*, 20(1), 63–73.

Bolger, A. M., Lohse, M., & Usadel, B. (2014). Trimmomatic: a flexible trimmer for Illumina sequence data. *Bioinformatics*, 30(15), 2114–2120.

Csilléry, K., François, O., & Blum, M. G. (2012). abc: an R package for approximate Bayesian computation (ABC). *Methods in Ecology and Evolution*, 3(3), 475–479.

Estoup, A., Beaumont, M., Sennedot, F., Moritz, C., & Cornuet, J. M. (2004). Genetic analysis of complex demographic scenarios: spatially expanding populations of the cane toad, *Bufo marinus*. *Evolution*, 58(9), 2021–2036.

Fu, L., Niu, B., Zhu, Z., Wu, S., & Li, W. (2012). CD-HIT: accelerated for clustering the next-generation sequencing data. *Bioinformatics*, 28(23), 3150–3152.

Fraïsse, C., Roux, C., Welch, J. J., & Bierne, N. (2014). Gene flow in a mosaic hybrid zone: is local introgression adaptive? *Genetics*, 197, 939-951.

Gérard, K., Bierne, N., Borsa, P., Chenuil, A., & Féral, J.-P. (2008). Pleistocene separation of mitochondrial lineages of *Mytilus spp*. mussels from Northern and Southern Hemispheres and strong genetic differentiation among southern populations. *Molecular Phylogenetics and Evolution*, 49(1), 84–91.

Grabherr, M. G., Haas, B. J., Yassour, M., Levin, J. Z., Thompson, D. A., Amit, I., Adiconis, X., Fan, L., Raychowdhury, R., & Zeng, Q. (2011). Full-length transcriptome assembly from RNA-Seq data without a reference genome. *Nature Biotechnology*, 29(7), 644.

Haas, B. J., Papanicolaou, A., Yassour, M., Grabherr, M., Blood, P. D., Bowden, J., Couger, M. B., Eccles, D., Li, B., & Lieber, M. (2013). De novo transcript sequence reconstruction from RNA-seq using the Trinity platform for reference generation and analysis. *Nature Protocols*, 8(8), 1494.

Hamilton, G., Stoneking, M., & Excoffier, L. (2005). Molecular analysis reveals tighter social regulation of immigration in patrilocal populations than in matrilocal populations. *Proceedings of the National Academy of Sciences*, 102(21), 7476–7480.

Hilbish, T. J., Mullinax, A., Dolven, S. I., Meyer, A., Koehn, R. K., & Rawson, P. D. (2000). Origin of the antitropical distribution pattern in marine mussels (*Mytilus spp.*): routes and timing of transequatorial migration. *Marine Biology*, 136(1), 69–77.

Huang, X., & Madan, A. (1999). CAP3: A DNA sequence assembly program. *Genome Research*, 9(9), 868–877.

Li, W., & Godzik, A. (2006). Cd-hit: a fast program for clustering and comparing large sets of protein or nucleotide sequences. *Bioinformatics*, 22(13), 1658–1659.

Lindberg, D. R. (1991). Marine biotic interchange between the northern and southern hemispheres. *Paleobiology*, 17(3), 308–324.

Magoč, T., & Salzberg, S. L. (2011). FLASH: fast length adjustment of short reads to improve genome assemblies. *Bioinformatics*, 27(21), 2957–2963.

Ripley, B., Venables, W., & Ripley, M. B. (2016). Package “nnet.” R Package Version, 7–3.

Roux, C., Tsagkogeorga, G., Bierne, N., & Galtier, N. (2013). Crossing the species barrier: genomic hotspots of introgression between two highly divergent *Ciona intestinalis* species. *Molecular Biology and Evolution,* 30(7), 1574–1587.

Roux, C., Fraïsse, C., Castric, V., Vekemans, X., Pogson, G. H., & Bierne, N. (2014). Can we continue to neglect genomic variation in introgression rates when inferring the history of speciation? A case study in a *Mytilus* hybrid zone. *Journal of Evolutionary Biology*, 27(8), 1662–1675.

Roux, C., Fraïsse, C., Romiguier, J., Anciaux, Y., Galtier, N., & Bierne, N. (2016). Shedding light on the grey zone of speciation along a continuum of genomic divergence. *PLoS Biology*, 14(12), e2000234.

Ross-Ibarra, J., Wright, S. I., Foxe, J. P., Kawabe, A., DeRose-Wilson, L., Gos, G., et al. (2008). Patterns of polymorphism and demographic history in natural populations of *Arabidopsis lyrata*. *PLoS ONE*, 3(6), e2411.

Rougemont, Q., & Bernatchez, L. (2018). The demographic history of Atlantic Salmon (*Salmo salar*) across its distribution range reconstructed from Approximate Bayesian Computations. *Evolution*, 72(6), 1261-1277

Tine, M., Kuhl, H., Gagnaire, P.-A., Louro, B., Desmarais, E., Martins, R. S., et al. (2014). European sea bass genome and its variation provide insights into adaptation to euryhalinity and speciation. *Nature Communications*, 5, 5770.

Table S1. Summary of prior distribution lower and upper bounds for 13 parameters (for each demographic model) shown in generation units (generation time=2 years). Effective populations sizes of derived (N_i , N_j) and ancestral ($N_{ancestral}$) populations; migration rate (*m*), where m_{ij} is the proportion of migrants from population *j* into population *i*; ancient migration rate (*ma*); time of secondary contact (T_{sc}); time of the onset of ancient gene flow (T_{nc}; backwards in time); divergence time (T_{div}).

	Demographic Model Priors: Lower bound – Upper bound					
**Parameter**	***pan***	***div***	***im***	***divSC***	***divAGF***	***divAGFSC***
N_i N_j $N_{ancestral}$	1000-500000	1000-500000	1000-500000	1000-500000	1000-500000	1000-500000
m_{ij}	-	-	0-0.0001	0-0.5	-	0-0.5
m_{ji}	-	-	0-0.0001	0	-	0
m_{aij}	-	-	-	-	0-0.0001	0-0.0001
m_{aji}	-	-	-	-	0-0.0001	0-0.0001
T_{sc}	-	-	-	5-300	-	5-300
T_{nc}	-	-	-	-	10000-1750000	10000-1750000
T_{div}	-	100000-1750000	100000-1750000	100000-1750000	100000-1750000	100000-1750000
ri	-	-	-	0.01-0.99	-	0.01-0.99
mu	0.00000002763	0.00000002763	0.00000002763	0.00000002763	0.00000002763	0.00000002763
rho	0.5	0.5	0.5	0.5	0.5	0.5

Table S2. Summary of stepwise comparison Akaike Information Criterion (AIC) between sequential migration models in TreeMix. We did not consider additional migration events when the difference between nested models was less than two (∆AIC < 2).

Number of migration events	Ln(likelihood)	AIC value	∆ AIC
0	-1552.53	3109.06	-
1	-361.959	727.918	2381.142
2	174.862	-345.724	1073.642
3	215.341	-426.682	80.958
4	218.552	-433.104	6.422
5	219.126	-434.252	1.148
6	219.483	-434.966	0.714
7	219.483	-434.966	0
8	219.483	-434.966	0
9	219.483	-434.966	0
10	219.483	-434.966	0

Table S3. Summary of model validation using pseudo-observed datasets. Values are shown for the A) Mediterranean-Tasmania and B) Atlantic-Tasmania populations pairs. Values for the best inferred model is indicated in **bold.**

A)

Model	Precision	Misclassification rate (Type I error)	Mean Type II error
pan	1.000	0.000	0.145
div	0.618	0.382	0.0776
ima	0.933	0.067	0.0088
divSC	0.390	0.610	0.0678
divAGF	**0.607**	**0.393**	**0.0826**
divSCAGF	0.364	0.636	0.0354

B)

Model	Precision	Misclassification rate (Type I error)	Mean Type II error
pan	1.000	0.000	0.1408
div	0.708	0.292	0.812
ima	0.948	0.052	0.0076
divSC	0.422	0.578	0.0618
divAGF	**0.587**	**0.413**	**0.0614**
divSCAGF	0.397	0.603	0.0348

Table S4. Summary of demographic model selection under an approximate Bayesian computation framework. Model posterior probabilities comparing all homogeneous and heterogeneous models (accounting for variation in N_e | *m* parameters) under each demographic scenario independently. **Bold** indicates the highest probability model for each comparison at an acceptance threshold of 0.001. Divergence time parameters were estimated under the best inferred demographic scenario (*divAGF*).

**Population**			**Demographic Model Probability: Proportion of accepted simulations**					
Mediterranean-Tasmania	**Model**	**Method**	**homo|homo**	**homo|hetero1**	**hetero1|homo**	**hetero1|hetero1**	**hetero2|homo**	**hetero2|hetero1**
	div	Neural Net	0.0037	-	0.0761	-	**0.4728**	-
	ima	Neural Net	0.0023	0.0471	0.0014	0.0825	0.0030	**0.8637**
	divSC	Neural Net	0.0191	0.1048	0.0196	0.1491	0.0199	**0.6875**
	divAGF	Neural Net	0.0013	0.0020	0.0259	0.0421	**0.6410**	0.2877
	divAGFSC	Neural Net	0.0217	0.0984	0.0201	0.2688	0.0246	**0.5664**
Atlantic-Tasmania								
	div	Neural Net	0.0001	-	0.0500	-	**0.9498**	**-**
	ima	Neural Net	0.0009	0.0167	0.0016	0.0395	0.0042	**0.9369**
	divSC	Neural Net	0.0177	0.1129	0.0130	0.0674	0.0405	**0.7486**
	divAGF	Neural Net	0.0040	0.0398	0.0118	0.0265	0.4291	**0.4888**
	divAGFSC	Neural Net	0.0222	0.0974	0.0192	0.1168	0.0465	**0.6979**

Figure S1. Summary of genomic data filtering schemes applied to respective analyses.

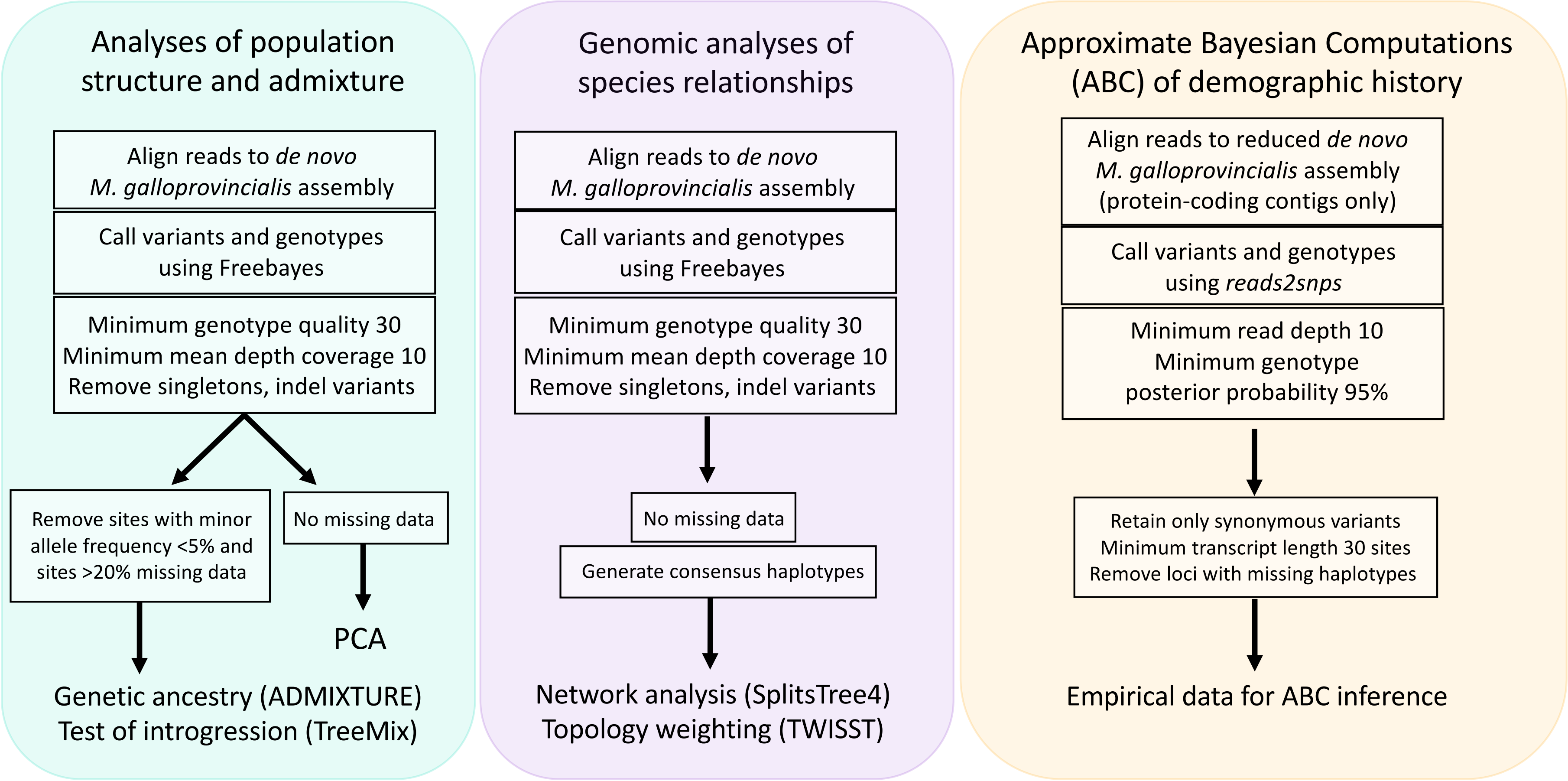

Figure S2. ADMIXTURE analyses for K=3 genetic clusters performed using 1 SNP per contig to account for linkage effects. Each bar represents an individual belonging to one or more ancestral clusters, corresponding to different colours.

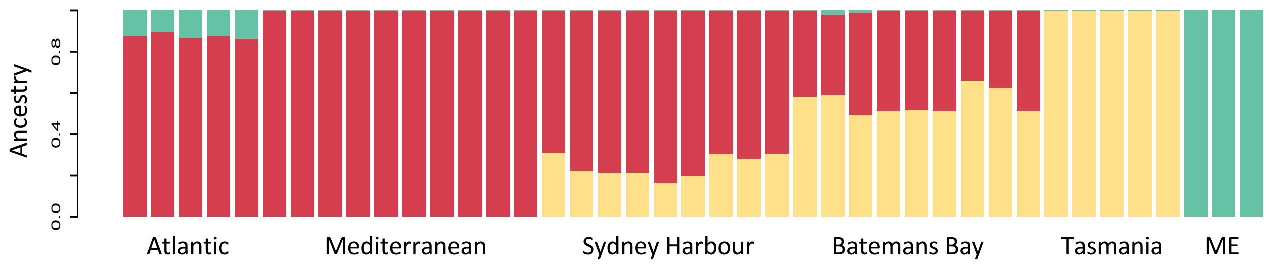

Figure S3. Maximum likelihood population tree without migration generated in TreeMix. The drift parameter indicates the amount of genetic drift that separates groups. Under a model of zero migration, the heat colours indicate pairwise population residual allele frequency covariance. The darkest boxes in this residual matrix indicate high genotypic covariance between Sydney Harbour or Batemans Bay populations with northern taxa.

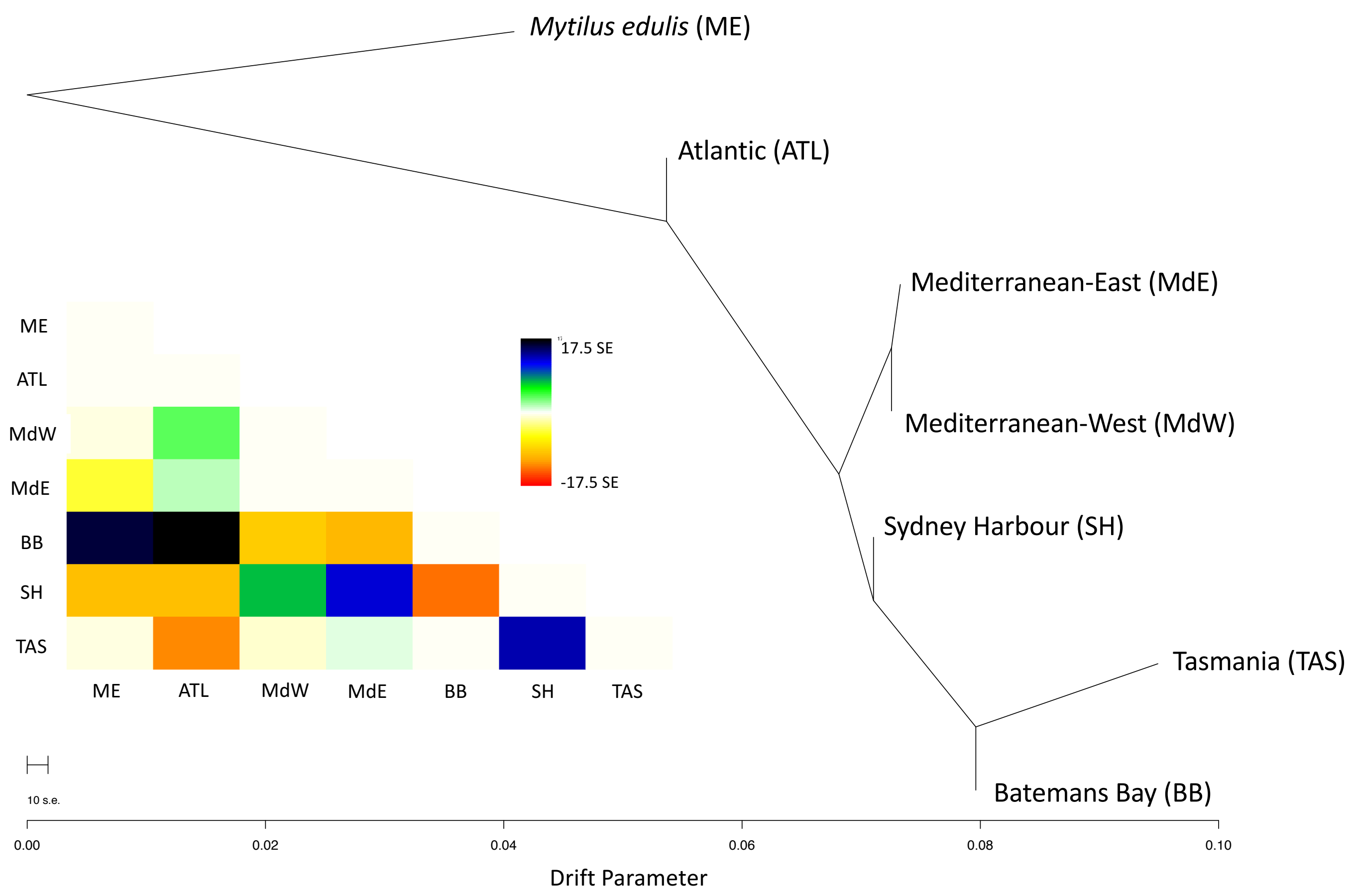

Figure S4. Summary of TWISST analyses of species relationships. Contributions of three possible unrooted topologies (hypotheses) to the nuclear species tree grouping *M. planulatus* (MP, Tasmania) with either *M. trossulus* (MT), *M. edulis* (ME) or *M. galloprovincialis* (MG, Mediterranean). Plots indicate i) mean topology contributions of 343 genealogies with a minimum tree length of 0.025; and ii) Distributions of the proportion of topology contributions across loci for three tested topologies**.**

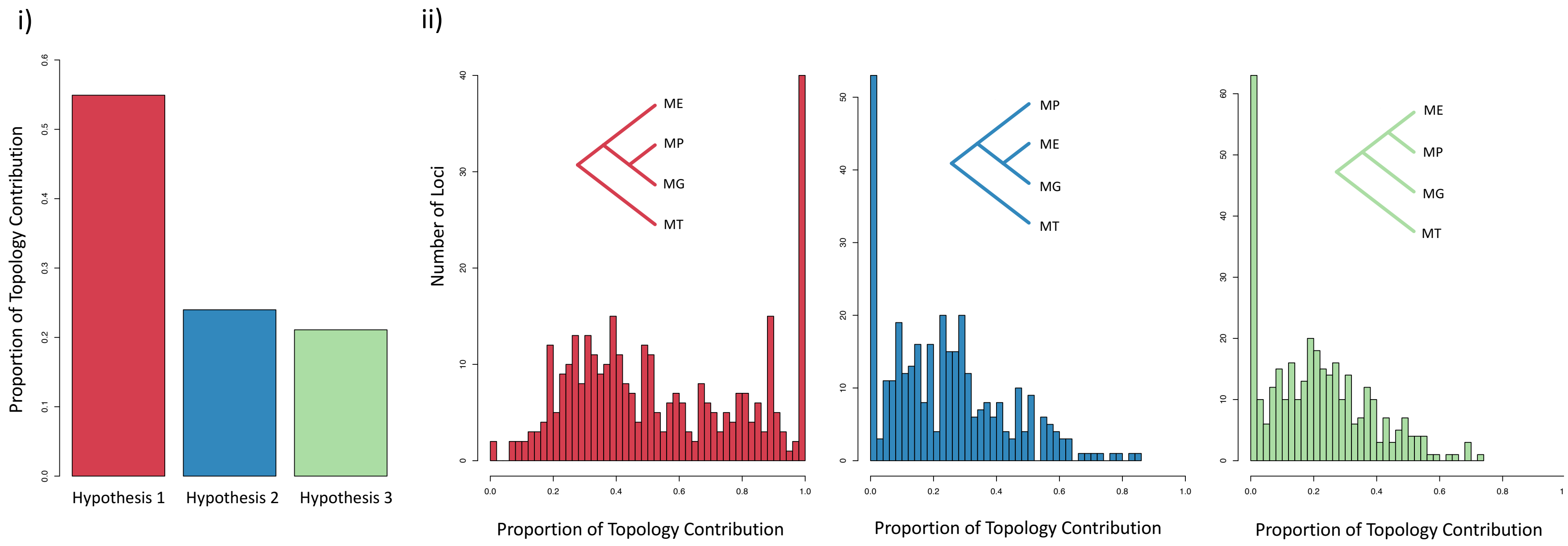

Figure S5. Summary of model choice validation using pseudo-observed datasets (PODS). Plots indicate the distributions of posterior probabilities for each true model. Model comparisons were carried out using 1000 PODS generated under the same model (shown in pink box plot).

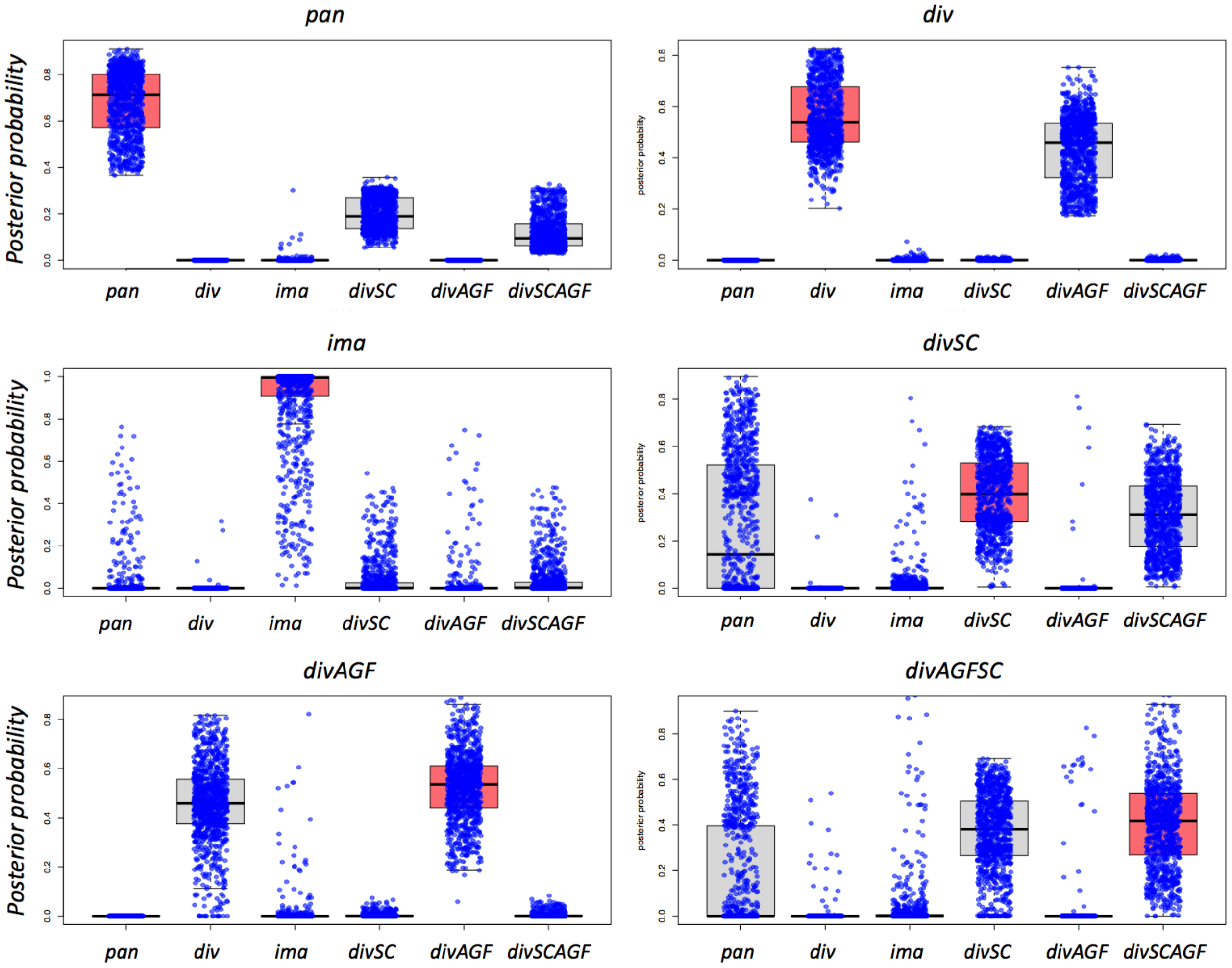

Supplement: Supplementary file 1 [file EVA-13-515-s001.pdf]
